# Ultrarapid Delayed Rectifier K^+^ Channelopathies in Human Induced Pluripotent Stem Cell-Derived Cardiomyocytes

**DOI:** 10.3389/fcell.2020.00536

**Published:** 2020-07-28

**Authors:** Sarah Hilderink, Harsha D. Devalla, Leontien Bosch, Ronald Wilders, Arie O. Verkerk

**Affiliations:** ^1^Department of Medical Biology, Amsterdam UMC, University of Amsterdam, Amsterdam, Netherlands; ^2^Department of Experimental Cardiology, Amsterdam UMC, University of Amsterdam, Amsterdam, Netherlands

**Keywords:** atrial fibrillation, cardiac differentiation, dynamic clamp, human pluripotent stem cells, ion channels, *KCNA5*, K_V_1.5, ultrarapid delayed rectifier potassium current

## Abstract

Atrial fibrillation (AF) is the most common cardiac arrhythmia. About 5–15% of AF patients have a mutation in a cardiac gene, including mutations in *KCNA5*, encoding the K_v_1.5 α-subunit of the ion channel carrying the atrial-specific ultrarapid delayed rectifier K^+^ current (I_Kur_). Both loss-of-function and gain-of-function AF-related mutations in *KCNA5* are known, but their effects on action potentials (APs) of human cardiomyocytes have been poorly studied. Here, we assessed the effects of wild-type and mutant I_Kur_ on APs of human induced pluripotent stem cell-derived cardiomyocytes (hiPSC-CMs). We found that atrial-like hiPSC-CMs, generated by a retinoic acid-based differentiation protocol, have APs with faster repolarization compared to ventricular-like hiPSC-CMs, resulting in shorter APs with a lower AP plateau. Native I_Kur_, measured as current sensitive to 50 μM 4-aminopyridine, was 1.88 ± 0.49 (mean ± SEM, *n* = 17) and 0.26 ± 0.26 pA/pF (*n* = 17) in atrial- and ventricular-like hiPSC-CMs, respectively. In both atrial- and ventricular-like hiPSC-CMs, I_Kur_ blockade had minimal effects on AP parameters. Next, we used dynamic clamp to inject various amounts of a virtual I_Kur_, with characteristics as in freshly isolated human atrial myocytes, into 11 atrial-like and 10 ventricular-like hiPSC-CMs, in which native I_Kur_ was blocked. Injection of I_Kur_ with 100% density shortened the APs, with its effect being strongest on the AP duration at 20% repolarization (APD_20_) of atrial-like hiPSC-CMs. At I_Kur_ densities < 100% (compared to 100%), simulating loss-of-function mutations, significant AP prolongation and raise of plateau were observed. At I_Kur_ densities > 100%, simulating gain-of-function mutations, APD_20_ was decreased in both atrial- and ventricular-like hiPSC-CMs, but only upon a strong increase in I_Kur_. In ventricular-like hiPSC-CMs, lowering of the plateau resulted in AP shortening. We conclude that a decrease in I_Kur_, mimicking loss-of-function mutations, has a stronger effect on the AP of hiPSC-CMs than an increase, mimicking gain-of-function mutations, whereas in ventricular-like hiPSC-CMs such increase results in AP shortening, causing their AP morphology to become more atrial-like. Effects of native I_Kur_ modulation on atrial-like hiPSC-CMs are less pronounced than effects of virtual I_Kur_ injection because I_Kur_ density of atrial-like hiPSC-CMs is substantially smaller than that of freshly isolated human atrial myocytes.

## Introduction

Worldwide, the prevalence of atrial fibrillation (AF) is around 1–2% (Potpara and Lip, 2011). Mutations in cardiac genes account for onset of 5–15% of AF cases (Darbar et al., 2003; Potpara and Lip, 2011). Mutations in *KCNA5* are associated with AF, although rare (Feghaly et al., 2018). *KCNA5* encodes the pore-forming α-subunit K_v_1.5 of the channel carrying the ultrarapid delayed rectifier K^+^ current (I_Kur_) (Fedida et al., 1993; Wang et al., 1993). In the human heart, K_v_1.5 and the mRNA encoding K_v_1.5 are both highly expressed in the atria (Ellinghaus et al., 2005; Gaborit et al., 2007), whereas expression of K_v_1.5 is very low in both endocardial and epicardial ventricular tissue (Mays et al., 1995; Gaborit et al., 2007) and expression of mRNA encoding K_v_1.5 is also low (Kääb et al., 1998; Gaborit et al., 2007). Accordingly, in their voltage clamp experiments on isolated human atrial and subepicardial ventricular myocytes, Amos et al. (1996) could not observe an I_Kur_-like current in their ventricular myocytes, in contrast to their atrial myocytes. I_Kur_ activates rapidly upon depolarizations to membrane potentials positive to −50 mV and is responsible for the early repolarization in human atrial action potentials (APs) (Wang et al., 1993; Amos et al., 1996; Wettwer et al., 2004; Li et al., 2008).

Both loss-of-function and gain-of-function mutations in *KCNA5* have been identified in patients with AF (Olson et al., 2006; Yang et al., 2009, 2010; Christophersen et al., 2013; Hayashi et al., 2015; Tian et al., 2015). Loss-of-function mutations in *KCNA5* are supposed to increase susceptibility to AF by prolonging the AP duration (APD) of atrial myocytes, which may eventually result in early afterdepolarizations (EADs) (Yang et al., 2009; Hayashi et al., 2015). Indeed, *in vitro* electrophysiological studies where I_Kur_ was blocked, representing complete *KCNA5* loss-of-function mutations, resulted in prolonged APDs and presence of EADs (Olson et al., 2006). EADs as a consequence of prolonged APDs have also been observed in *in silico* studies on loss-of-function *KCNA5* mutations (Colman et al., 2017; Ni et al., 2017).

Gain-of-function mutations, on the other hand, are presumed to cause AF by shortening the effective refractory period (ERP) of the atrial AP, facilitating re-entry wavelets in the atria (Nattel, 2002; Christophersen et al., 2013). This hypothesis is supported by *in silico* studies, which demonstrated that increased I_Kur_ density, representing gain-of-function mutations, resulted in a shortened APD and arrhythmogenesis in human atrial tissue (Colman et al., 2017; Ni et al., 2017).

Although the *in silico* studies are instrumental in determining the potential effect of both loss-of-function and gain-of-function mutations in *KCNA5*, detailed electrophysiological studies of the *KCNA5* mutations in human cardiomyocytes are limited. Human induced pluripotent stem cell cardiomyocytes (hiPSC-CMs) have become a highly suitable tool to study cardiac ion channelopathies and their electrophysiology (Zhang et al., 2011; Hoekstra et al., 2012; Verkerk et al., 2017). Over time, the technique of cardiomyocyte differentiation has advanced, facilitating the generation of distinct atrial- and ventricular-like hiPSC-CM populations (Zhang et al., 2011; Devalla et al., 2015; Devalla and Passier, 2018). In the present study, we employed dynamic clamp to investigate the effects of loss-of-function and gain-of-function mutations in *KCNA5* in both atrial- and ventricular-like hiPSC-CMs.

## Materials and Methods

### hiPSC-CM Differentiation

hiPSC-CMs were generated from the control LUMC0099iCTRL04 hiPSC line, which was derived from human fibroblasts extracted through skin biopsies from of a Caucasian woman. The LUMC0099iCTRL04 line is registered in the Human Pluripotent Stem Cell Registry (Seltmann et al., 2016), which contains all details pertaining to its generation and characterization (hPSCreg, 2019). hiPSC clones showing stem cell morphology were characterized for pluripotency marker expression and differentiation potential to hiPSC-CMs in BPEL medium (Ng et al., 2008) containing activin-A, BMP4, and CHIR99021 (Devalla et al., 2016). After 3 days, this medium was replaced by BPEL medium containing XAV939 (Tocris Biosciences) for ventricular differentiation (Ng et al., 2008; Devalla et al., 2016). To differentiate hiPSC-CMs to atrial-like hiPSC-CMs, 1 μM all-trans retinoic acid (RA) was added (Devalla et al., 2015). Twenty days after differentiation, hiPSC-CMs were dissociated with TrypLE Select (Life Technologies), and plated at a low density (≈7.5 × 10^4^ cells) on Matrigel coated coverslips in BPEL medium (Devalla et al., 2016).

### Patch-Clamp Measurements

#### Data Acquisition

Electrophysiological recordings were performed 4–13 days post dissociation from spontaneously beating single hiPSC-CMs. RA-treated hiPSC-CMs displaying a short, pulse-like beating pattern and non-RA-treated hiPSC-CMs with a contraction-like beating pattern were selected for data acquisition. APs and I_Kur_ were recorded at 36–37°C with the perforated patch-clamp technique using an Axopatch 200B amplifier (Molecular Devices, Sunnyvale, CA, United States). Data acquisition and analysis were performed with custom software. Signals were low-pass filtered with a cut-off frequency of 2 kHz and digitized at 40 and 5 kHz for AP and I_Kur_ recordings, respectively. Cell membrane capacitance (C_m_, in pF) was calculated by dividing the time constant of the decay of capacitive transient when hyperpolarized by 5 mV from −40 mV in voltage clamp by series resistance. C_m_ of atrial- and ventricular-like hiPSC-CMs was 16.4 ± 2.3 pF (mean ± SEM, *n* = 28), and 19.2 ± 2.5 pF (*n* = 27), respectively (*t*-test, N.S.). Patch pipettes with a resistance of ≈2.0 MΩ were pulled from borosilicate glass (Harvard Apparatus) and filled with solution containing (in mM): 125 K-gluconate, 20 KCl, 5 NaCl, 0.44 Amphotericin-B, 10 HEPES; pH set to 7.2 (KOH). Cells were superfused with modified Tyrode’s solution containing (in mM): 140 NaCl, 5.4 KCl, 1.8 CaCl_2_, 1.0 MgCl_2_, 5.5 D-glucose, 5 HEPES; pH set to 7.4 (NaOH). All potentials were corrected for the estimated liquid junction potential of −15 mV (Barry and Lynch, 1991).

#### Action Potential Recordings

APs were elicited at 1 Hz by 3-ms, ≈1.2 × threshold current pulses through the patch pipette. The AP parameters analyzed were resting membrane potential (RMP, in mV), maximum upstroke velocity (dV/dt_max_, in V/s), AP amplitude (APA, in mV), AP duration at 20, 50, and 90% repolarization (APD_20_, APD_50_, and APD_90_, respectively, in ms), and AP plateau amplitude (APPlatA, in mV), derived from the membrane potential (V_m_) at 50 ms after the time of dV/dt_max_.

#### Native I_Kur_ Recordings

Native I_Kur_ was activated by 200-ms voltage clamp steps from −50 to +50 mV. A 50-ms prepulse to 0 mV was applied to activate and inactivate remaining transient membrane currents. Series resistance was compensated by ≥ 80%. I_Kur_ was measured as the current sensitive to 50 μM 4-aminopyridine (4-AP) (Wang et al., 1993; Caballero et al., 2010), and was normalized to C_m_ to calculate current density (in pA/pF).

#### Dynamic Clamp

Although inward rectifier K^+^ current (I_K__1_) is not necessarily low in hiPSC-CMs (Horváth et al., 2018), hiPSC-CMs tend to lack I_K__1_, which is responsible for stabilizing the RMP of atrial and ventricular myocytes, and thus show spontaneous activity (Dhamoon and Jalife, 2005; Hoekstra et al., 2012; Verkerk et al., 2017). The RMP of our atrial- and ventricular-like hiPSC-CMs was stabilized and set at a regular hyperpolarized value using the dynamic clamp technique (Wilders, 2006). A virtual Kir2.1-based I_K__1_, with a standard peak current density of 2 pA/pF, was injected into the hiPSC-CMs and this I_K__1_ was computed in real time, based on the acquired V_m_, following the approach of Meijer van Putten et al. (2015). Accordingly, the mathematical equation for I_K__1_ reads

$$I_{\mathrm{K1}}=0.12979\times \left(\frac{V_{\text{m}}-E_{\text{K}}}{1.0+e^{\left(0.093633\times \left(V_{\text{m}}+72\right)\right)}}\right)$$

In this equation, in which the rectification properties of I_K__1_ are implemented through a Boltzmann equation, I_K__1_ is in pA/pF and V_m_ is in mV. E_K_ is the Nernst potential for potassium, which amounts to −86.9 mV in our experimental setting.

**FIGURE 1:**
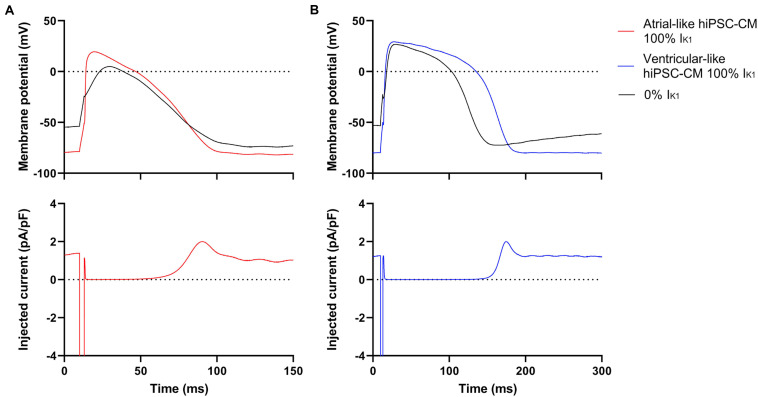
Typical action potentials (APs) of atrial- and ventricular-like hiPSC-CM with and without I_K__1_ injection. **(A)** Superimposed APs (top panel) of an atrial-like hiPSC-CM in absence (black line) and presence (red line) of 100% virtual I_K__1_ injected through dynamic clamp and associated injected current (bottom panel) consisting of stimulus current and 0% or 100% virtual I_K__1_. **(B)** Superimposed APs and associated injected current of a ventricular-like hiPSC-CM. APs elicited at 1 Hz by a 3-ms stimulus of 100 pA. Note difference in time scale between panels **(A,B)**.

The effect of the injection of this virtual I_K__1_ is illustrated in Figure 1, which shows the APs of typical atrial-like and ventricular-like hiPSC-CMs in the absence and presence of this virtual I_K__1_ (top panels) and the associated injected current (bottom panels), which consists of this I_K__1_ and a short inward stimulus current. A virtual Kir2.1-based I_K__1_, characteristic for human ventricular myocytes (Wang et al., 1998), was used in both atrial- and ventricular-like hiPSC-CMs because a more ‘atrial-like’ I_K__1_ in hiPSC-CMs results in a substantial current during early repolarization due to its reduced rectification (Meijer van Putten et al., 2015; Verkerk et al., 2017; Fabbri et al., 2019), and we wanted to prevent a prominent overlap and potential interference of I_K__1_ and I_Kur_ during the course of an action potential.

**FIGURE 2:**
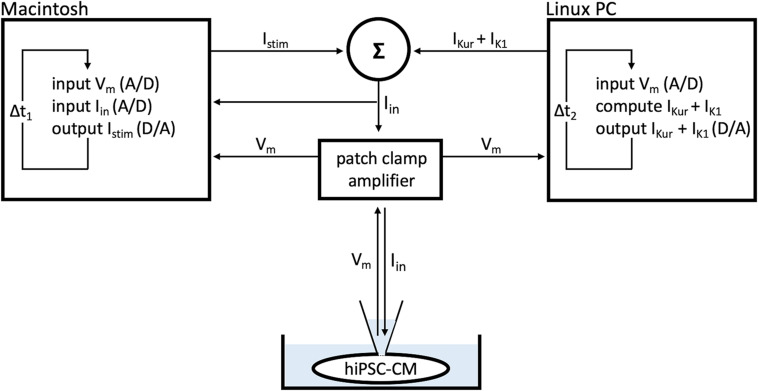
Dynamic clamp setup. Ultrarapid delayed rectifier K^+^ current (I_Kur_) and inward rectifier potassium current (I_K__1_) were computed in real time on a PC running the Linux operating system and Real-Time Experiment Interface (RTXI) software (Patel et al., 2017), based on the acquired membrane potential (V_m_) of the human induced pluripotent stem cell-derived cardiomyocyte (hiPSC-CM). Data were recorded on an Apple Macintosh computer using custom software to visualize and control the experiment. The sum of the stimulus current (I_stim_), I_K__1_, and I_Kur_ resulted in the current that was injected into the hiPSC-CM (I_in_). Sample rates were 30 kHz (Δt_1_ = 33.33 μs) and 20 kHz (Δt_2_ = 50 μs).

The dynamic clamp technique was also used to provide our atrial- and ventricular-like hiPSC-CMs with a virtual wild-type or mutant I_Kur_, as illustrated in Figure 2. Like I_K__1_, I_Kur_ was computed in real time, based on the acquired value of V_m_. I_Kur_ was formulated as detailed in Section “I_Kur_ Equations” below. Virtual I_Kur_ was injected into atrial- and ventricular-like hiPSC-CMs with a fully activated conductance of 12.5, 25, 50, and 75% of its wild-type value to mimic loss-of-function mutations, and 125, 150, 175, and 200% of its wild-type value to mimic gain-of-function mutations.

#### I_Kur_ Equations

To compute I_Kur_ in our dynamic clamp system, I_Kur_ equations of the comprehensive human atrial myocyte model by Maleckar et al. (2009) were used. These equations were also adopted by Grandi et al. (2011) in their human atrial action potential and Ca^2+^ model and read:

$$I_{\mathrm{Kur}}=g_{\mathrm{Kur}}\times a_{\mathrm{ur}}\times i_{\mathrm{ur}}\times \left(V-E_{K}\right)$$

$${\mathrm{da}}_{\mathrm{ur}}/\mathrm{dt}=\left(a_{\mathrm{ur},\infty }-a_{\mathrm{ur}}\right)/\tau_{\mathrm{aur}}$$

$${\mathrm{di}}_{\mathrm{ur}}/\mathrm{dt}=\left(i_{\mathrm{ur},\infty }-i_{\mathrm{ur}}\right)/\tau_{\mathrm{iur}}$$

$$a_{\mathrm{ur},\infty }=\frac{1.0}{\left[1.0+e^{-\left(V+\frac{6.0}{8.6}\right)}\right]}$$

$$i_{\mathrm{ur},\infty }=\frac{1.0}{\left[1.0+e^{\left(V+\frac{7.5}{10.0}\right)}\right]}$$

$$\tau_{a\,u\,r}=\frac{0.009}{\left[1.0+e^{\left(V+\frac{5.0}{12.0}\right)}\right]}+0.0005$$

$$\tau_{i\,u\,r}=\frac{0.59}{\left[1.0+e^{\left(V+\frac{60.0}{10.0}\right)}\right]}+3.05$$

In these equations, the dimensionless Hodgkin and Huxley-type activation and inactivation gating variables, ranging between 0 and 1, are denoted by a_ur_ and i_ur_, respectively, whereas I_Kur_ (in pA/pF), g_Kur_ (in nS/pF), V (in mV), E_K_ (in mV), and t (in s) denote the ultrarapid delayed rectifier outward K^+^ current, its fully activated conductance, the membrane potential, the K^+^ reversal potential, and the time, respectively. The steady-state values of a_ur_ and i_ur_ are denoted by a_ur,__∞_ and i_ur,__∞_, respectively, and the associated time constants by τ_aur_ (in s) and τ_iur_ (in s), respectively. As in the models by Maleckar et al. (2009) and Grandi et al. (2011), a default value of 0.045 nS/pF was used for g_Kur_. Of note, Maleckar et al. (2009) based this value on experimental data on I_Kur_ density in human atrial myocytes.

### Statistical Analysis

Data are presented as mean ± SEM. Statistical analysis was carried out with SigmaStat 3.5 software (Systat Software, Inc., San Jose, CA, United States). Native I_Kur_ density of atrial- and ventricular-like hiPSC-CMs was compared with an independent samples *t*-test. Two-way repeated measures ANOVA followed by the Student–Newman–Keuls *post hoc* test was used for comparing AP parameters of atrial- and ventricular-like hiPSC-CMs in absence or presence of 4-AP. One-way repeated measures ANOVA followed by the Student–Newman–Keuls *post hoc* test was used for comparing the effect of injecting virtual I_Kur_ at various densities into atrial- and ventricular-like hiPSC-CMs. *P* < 0.05 was considered statistically significant.

## Results

### Atrial- and Ventricular-Like hiPSC-CM APs

**FIGURE 3:**
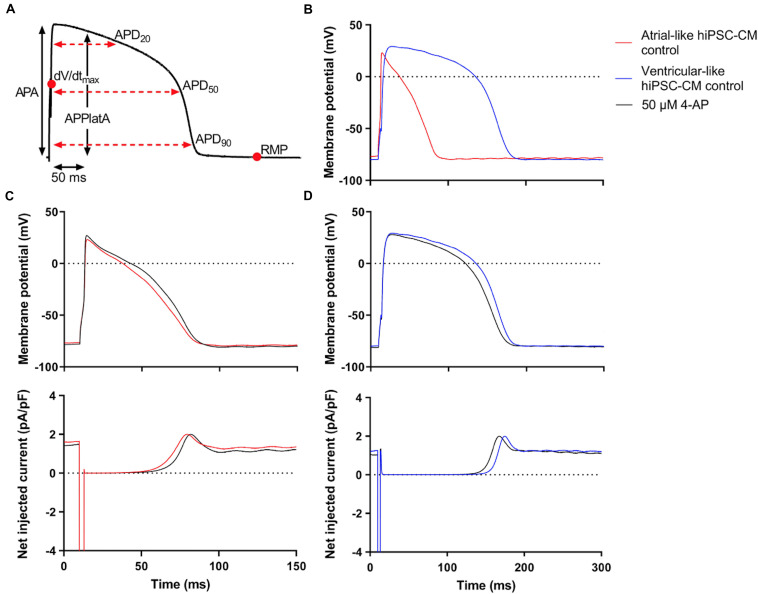
Control atrial- and ventricular-like hiPSC-CM APs. **(A)** AP parameters used for analysis: resting membrane potential (RMP), maximum upstroke velocity (dV/dt_max_), AP amplitude (APA), AP duration at 20, 50, and 90% repolarization (APD_20_, APD_50_, and APD_90_, respectively), and AP plateau amplitude at 50 ms after reaching dV/dt_max_ (APPlatA). **(B)** Superimposed typical APs of an atrial-like hiPSC-CM (red line) and a ventricular-like hiPSC-CM (blue line). **(C)** Superimposed APs (top panel) and associated net current consisting of stimulus current and virtual I_K__1_ injected through dynamic clamp (bottom panel) of an atrial-like hiPSC-CM in absence (red line) and presence of 50 μM 4-aminopyridine (4-AP) (black line). **(D)** Superimposed APs (top panel) and associated net injected current (bottom panel) of a ventricular-like hiPSC-CM. APs elicited at 1 Hz by a 3-ms stimulus of 100 pA. Note difference in time scale between panels **(C,D)**.

APs were recorded from single atrial- and ventricular-like hiPSC-CMs that showed spontaneous beating upon visual inspection, clearly indicating a healthy and myocardial status. APs were elicited at 1 Hz and virtual I_K__1_ was injected into the cells, based on the approach of Meijer van Putten et al. (2015), to stabilize the RMP and set it at a regular hyperpolarized value. Figure 3B shows typical atrial- and ventricular-like hiPSC-CM APs. AP parameters, as illustrated in Figure 3A, are summarized in Table 1. Atrial-like hiPSC-CMs repolarize faster than ventricular-like APs, resulting in a significantly shorter APD_20_, APD_50_, and APD_90_. The ventricular-like APs have a prominent plateau phase at relatively positive potentials, in contrast with the atrial-like hiPSC-CMs that show a less prominent plateau phase at less positive potentials, if any plateau at all. Consequently, APPlatA was significantly smaller in atrial-like hiPSC-CMs and thus appeared a strong tool to distinguish between atrial-like and ventricular-like hiPSC-CMs. APA and dV/dt_max_ did not differ between the atrial- and ventricular-like hiPSC-CMs, but RMP was less negative in ventricular-like hiPSC-CMs.

**TABLE 1:** AP parameters of atrial- and ventricular-like hiPSC-CMs in absence and presence of 4-AP.

	Atrial-like hiPSC-CMs (*n* = 11)		Ventricular-like hiPSC-CMs (*n* = 10)	
	Baseline	4-AP	Baseline	4-AP
RMP (mV)	−81.31 ± 0.43	−80.83 ± 0.65	−75.52 ± 1.69*	−74.92 ± 1.71^#^
dV/dt_max_ (V/s)	74.54 ± 13.10	76.47 ± 13.41	83.70 ± 20.21	94.33 ± 24.43
APA (mV)	96.61 ± 2.66	98.33 ± 1.44	105.61 ± 3.69	104.59 ± 3.88
APD_20_ (ms)	35.86 ± 4.35	38.13 ± 4.69	77.73 ± 11.59*	68.68 ± 10.67^‡#^
APD_50_ (ms)	60.99 ± 5.55	65.05 ± 5.38	127.93 ± 19.67*	119.41 ± 18.09^‡#^
APD_90_ (ms)	81.27 ± 6.37	86.76 ± 5.92^†^	155.01 ± 21.49*	147.68 ± 20.38^‡#^
APPlatA (mV)	53.61 ± 5.25	60.53 ± 4.53^†^	89.61 ± 4.51*	89.42 ± 4.30^#^

Data are AP parameters (mean ± SEM) of APs elicited at 1 Hz in absence (baseline) and presence of 50 μM 4-aminopyridine (4-AP). *P < 0.05 atrial- *vs*. ventricular-like hiPSC-CMs (baseline); ^†^*P* < 0.05 4-AP *vs*. baseline of atrial-like hiPSC-CMs; ^‡^*P* < 0.05 4-AP *vs*. baseline of ventricular-like hiPSC-CMs; ^#^*P* < 0.05 atrial- *vs*. ventricular-like hiPSC-CMs (4-AP).

Next, the cells were superfused with 50 μM 4-AP to block intrinsic I_Kur_. Figures 3C,D, top panels, show typical atrial- and ventricular-like hiPSC-CMs in absence (red and blue lines, respectively) and presence of 4-AP (black lines). The associated injected currents, which each consist of I_K__1_ and the 3-ms stimulus current applied at 10 ms, are displayed in the bottom panels of Figures 3C,D. In atrial-like hiPSC-CMs, I_Kur_ blockade resulted in a significantly increased APD_90_ and APPlatA, while other AP parameters were unaffected (Table 1). In contrast, in ventricular-like hiPSC-CMs, APD_20_, APD_50_ and APD_90_ were significantly decreased upon I_Kur_ blockade (Table 1). The small AP shortening likely results from a time effect rather than a drug effect because I_Kur_ is virtually absent in our ventricular-like hiPSC-CMs. Comparing atrial- with ventricular-like APs during I_Kur_ blockade, most AP parameters still differ significantly, except APA and dV/dt_max_.

### Native I_Kur_

**FIGURE 4:**
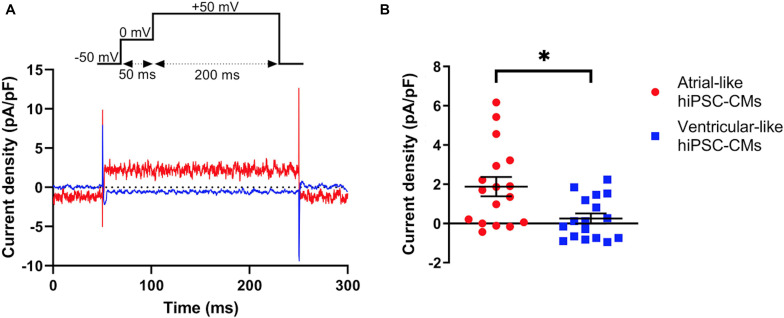
Density of I_Kur_ measured as 50 μM 4-AP-sensitive current. **(A)** Typical 4-AP-sensitive currents in an atrial- and a ventricular-like hiPSC-CM (red and blue traces, respectively) upon 200-ms depolarizing voltage clamp steps to +50 mV from a holding potential of −50 mV. **(B)** I_Kur_ density in 17 atrial- and 17 ventricular-like hiPSC-CMs. *P < 0.05.

Native I_Kur_ density in atrial- and ventricular-like hiPSC-CMs was quantified during 200-ms depolarizing voltage clamp steps as the current sensitive to 50 μM 4-AP. Figure 4A shows typical examples in an atrial-like (red trace) and a ventricular-like hiPSC-CM (blue trace). On average, I_Kur_ density in atrial-like hiPSC-CMs was significantly larger than in ventricular-like hiPSC-CMs, with densities of 1.88 ± 0.49 (*n* = 17) and 0.26 ± 0.26 (*n* = 17) pA/pF, respectively (Figure 4B).

If the voltage clamp protocol of Figure 4A is repeated in computer simulations with the Maleckar et al. (2009) human atrial myocyte model (Wilders, 2018), an I_Kur_ density of 5.45 pA/pF is obtained. We regard the latter as a realistic value for human atrial myocytes since Maleckar et al. (2009) based the characteristics of their model I_Kur_ on experimental data on I_Kur_ from isolated human atrial myocytes.

### Effects of Baseline Virtual I_Kur_ on APs of Atrial- and Ventricular-Like hiPSC-CMs

Next, we studied the effects of a virtual I_Kur_ on APs of atrial and ventricular-like hiPSC-CMs using dynamic clamp. In the human heart, I_Kur_ is highly atrial-specific (Ellinghaus et al., 2005; Gaborit et al., 2007). However, the dynamic clamp technique allowed us to inject a virtual I_Kur_ in both atrial- and ventricular-like hiPSC-CMs and thus assess to which extent this made their action potential morphology become similar. In either case, 4-AP (50 μM) was present to ensure that any native I_Kur_ was blocked (Wang et al., 1993) and from here on we name this condition 0% I_Kur_. First, we injected a virtual I_Kur_ as implemented in the Maleckar et al. (2009) human atrial myocyte model, i.e., with the aforementioned 5.45 pA/pF density at +50 mV, which we here consider as 100% density.

**FIGURE 5:**
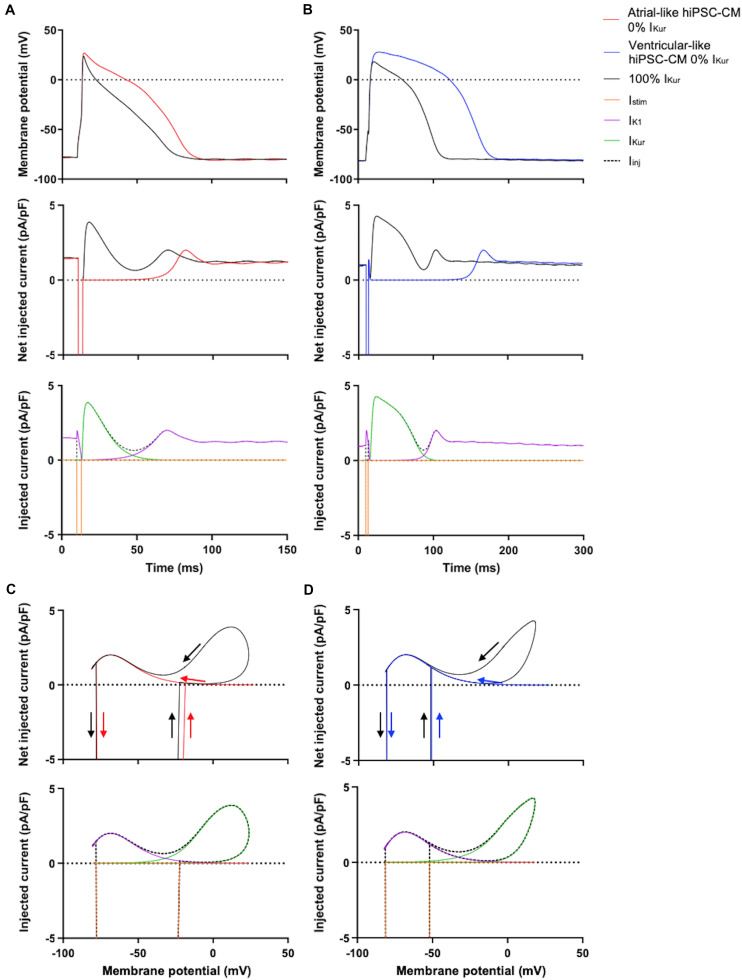
Effect of injection of 100% virtual I_Kur_ through dynamic clamp on APs of atrial- and ventricular-like hiPSC-CMs. **(A)** Superimposed APs (top panel) of an atrial-like hiPSC-CM at 0% (red line) and 100% virtual I_Kur_ (black line) and associated net injected current (middle panel), consisting of I_K__1_, 0% or 100% I_Kur_, and a short stimulus current, as shown in the bottom panel in case of 100% I_Kur_. **(B)** Superimposed APs (top panel), associated net injected current (middle panel), and its individual components in case of 100% I_Kur_ (bottom panel) of a ventricular-like hiPSC-CM. **(C,D)** Phase plane plots (top panel) of the action potentials and injected currents of panels **(A,B)** with their individual components in case of 100% I_Kur_ (bottom panel). APs elicited at 1 Hz by a 3-ms stimulus of 100 pA. Note difference in time scale between panels **(A,B)**.

**FIGURE 6:**
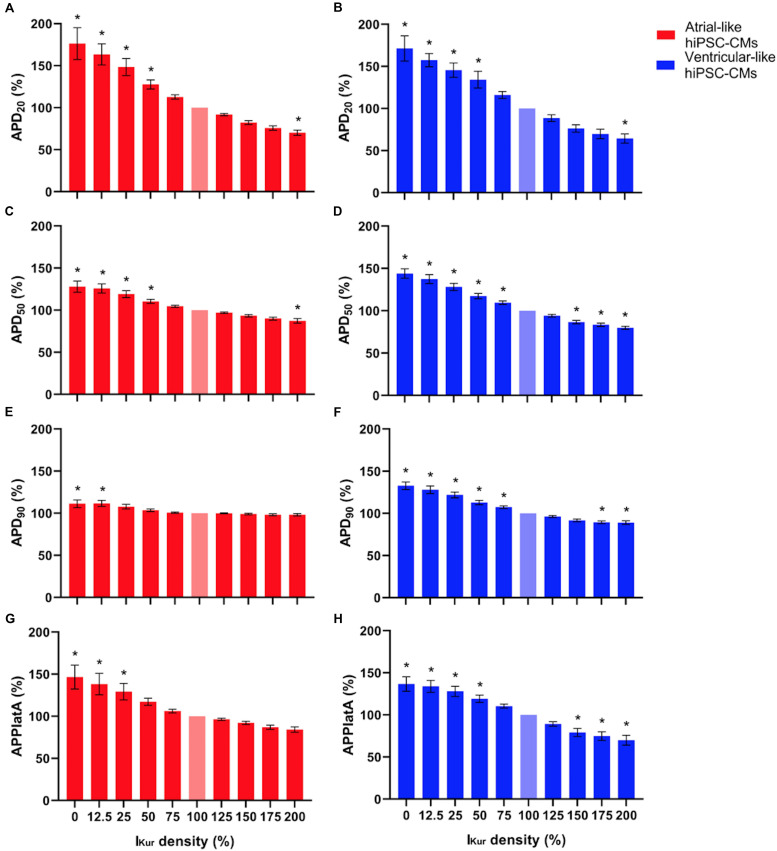
**(A,B)** APD_20_, **(C,D)** APD_50_, **(E,F)** APD_90_, and **(G,H)** APPlatA of atrial- and ventricular-like hiPSC-CMs (left and right panels, respectively) at virtual I_Kur_ densities ranging from 0 to 200%. AP parameters are expressed as percentage relative to their value at 100% I_Kur_ density (bleached bars). Data from 11 atrial- and 10 ventricular-like hiPSC-CMs. *P < 0.05.

**FIGURE 7:**
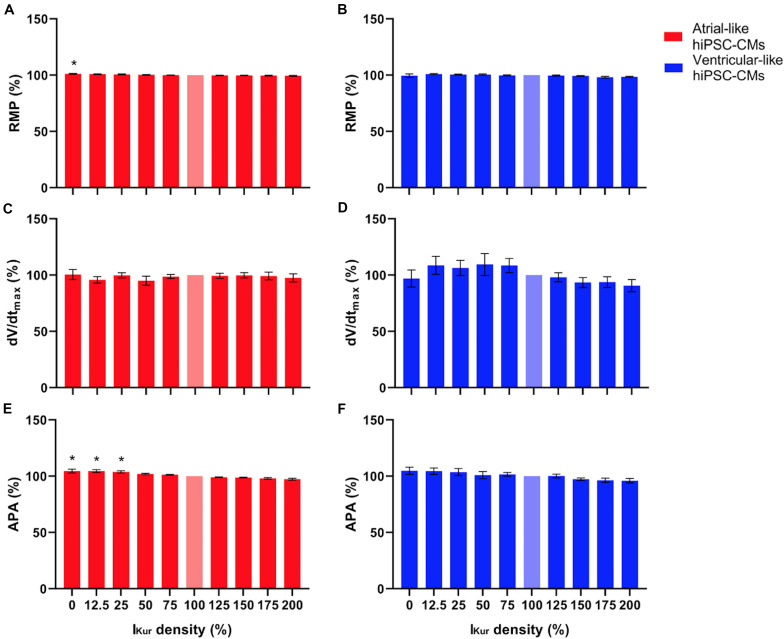
**(A,B)** RMP, **(C,D)** dV/dt_max_, and **(E,F)** APA of atrial- and ventricular-like hiPSC-CMs (left and right panels, respectively) at virtual I_Kur_ densities ranging from 0 to 200%. AP parameters are expressed as percentage relative to their value at 100% I_Kur_ density (bleached bars). Data from 11 atrial- and 10 ventricular-like hiPSC-CMs. *P < 0.05.

Figures 5A,B, top panels, show typical examples of APs recorded from atrial- and ventricular-like hiPSC-CM at 0% (red and blue lines, respectively) and 100% I_Kur_ (black lines). The injected current, which now consists of I_K__1_, 0% or 100% I_Kur_, and a short stimulus current, is shown in the middle panels of Figures 5A,B. The average effects on the AP parameters of 11 atrial- and 10 ventricular-like hiPSC-CMs appear as the bars at 0% and 100% I_Kur_ in Figures 6A–H, 7A–F, in which each of the AP parameters is expressed as a percentage of its value obtained at 100% I_Kur_. Injection of I_Kur_ shortened the AP of both atrial- and ventricular-like hiPSC-CMs, while the AP plateau was suppressed (Figures 5, 6). dV/dt_max_ was unaltered in both atrial-and ventricular-like hiPSC-CMs, but in atrial-like hiPSC-CMs RMP was significantly more negative and APA significantly larger in absence than in presence of I_Kur_ (Figure 7). The small 1.2% difference in RMP, equivalent to a 1.0-mV hyperpolarization, is likely a false positive because injection of various amounts of I_Kur_ did not affect the RMP in either atrial-like or ventricular-like hiPSC-CMs (see below).

The phase plane plots of Figures 5C,D show the injected currents of the middle panels of Figures 5A,B plotted against the associated membrane potentials of the APs shown in the top panels of Figures 5A,B. The start and the end of the negative depolarizing current that flows during the stimulus are indicated by downward and upward vertical arrows, respectively. The black loops of the phase plane plots clearly show that I_Kur_ is a repolarizing current that is already activated during the 3-ms stimulus and stays active until repolarization reaches −40 to −50 mV and the black traces ‘fuse’ with the red and blue traces of the action potentials without I_Kur_ (horizontal arrows). At these negative potentials, I_Kur_ becomes small because of both deactivation—rather than inactivation, which is much slower—and diminishing driving forces. The maximum I_Kur_ during the atrial-like AP is slightly larger compared to the ventricular-like AP because in this particular example the atrial-like AP reaches a higher peak than the ventricular-like AP, which results in a larger driving force for I_Kur_.

### Effects of I_Kur_ Loss-of-Function Mutations in Atrial- and Ventricular-Like hiPSC-CMs

**FIGURE 8:**
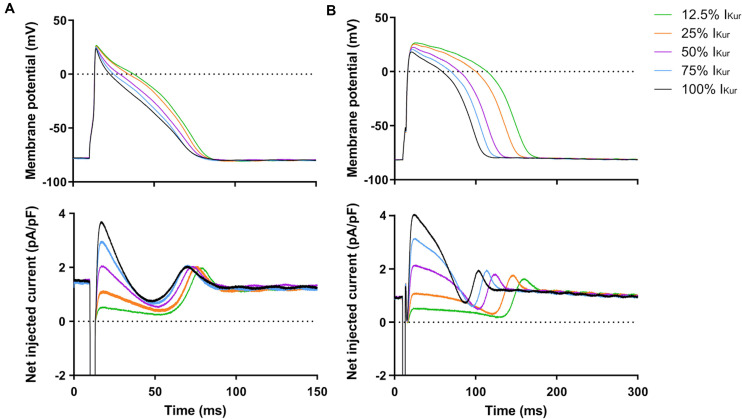
Effect of I_Kur_ loss-of-function mutations on APs of atrial- and ventricular-like hiPSC-CMs. **(A)** Superimposed APs of an atrial-like hiPSC-CM upon injection of 12.5–100% virtual I_Kur_ through dynamic clamp and associated injected current (bottom panel), consisting of I_K__1_, 12.5–100% I_Kur_, and a short stimulus current. **(B)** Superimposed APs (top panel) and associated injected current (bottom panel) of a ventricular-like hiPSC-CM. APs elicited at 1 Hz by a 3-ms stimulus of 100 pA. Note difference in time scale between panels **(A,B)**.

Next, we studied the effects of loss-of-function mutations in *KCNA5*, resulting in a decrease in I_Kur_. Therefore, we decreased the fully activated conductance of the virtual I_Kur_ conductance to 75, 50, 25, and 12.5% of its control value. Figure 8 shows typical examples of the effects on the APs of atrial- and ventricular-like hiPSC-CMs. The average changes in AP parameters are shown in Figures 6, 7. In both atrial- and ventricular-like hiPSC-CMs, APD_20_, APD_50_, and APD_90_ significantly increased upon a reduction in I_Kur_ (Figures 6A–F). However, while the increase in APD_90_ in atrial-like APs is only present upon severe I_Kur_ reduction, APD_90_ prolongation in ventricular-like APs is already present at a mild reduction (Figures 6E,F). APPlatA was significantly increased in both atrial- and ventricular-like APs (Figures 6G,H). RMP and dV/dt_max_ were unaffected (Figures 7A–D), whereas a slight increase in APA was observed, but only in atrial-like hiPSC-CMs at severe reductions of I_Kur_ (Figures 7E,F).

### Effects of I_Kur_ Gain-of-Function Mutations in Atrial- and Ventricular-Like hiPSC-CMs

**FIGURE 9:**
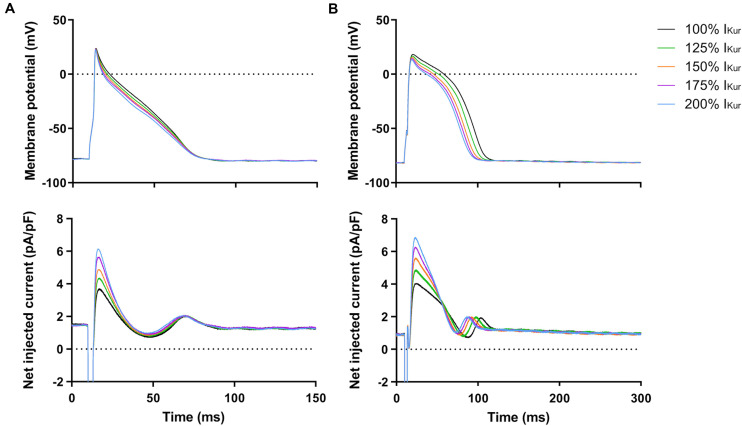
Effect of I_Kur_ gain-of-function mutations on APs of atrial- and ventricular-like hiPSC-CMs. **(A)** Superimposed APs of an atrial-like hiPSC-CM upon injection of 100–200% virtual I_Kur_ through dynamic clamp and associated injected current (bottom panel), consisting of I_K__1_, 100–200% I_Kur_, and a short stimulus current. **(B)** Superimposed APs (top panel) and associated injected current (bottom panel) of a ventricular-like hiPSC-CM. APs elicited at 1 Hz by a 3-ms stimulus of 100 pA. Note difference in time scale between panels **(A,B)**.

Finally, we studied the effects of gain-of-function mutations in *KCNA5*, resulting in an increase in I_Kur_. Therefore, we increased the fully activated conductance of the virtual I_Kur_ conductance to 125, 150, 175, and 200% of its control value. Figure 9 shows typical examples of the effects on the APs of atrial- and ventricular-like hiPSC-CMs. The average changes in AP parameters are shown in Figures 6, 7. In both atrial- and ventricular-like hiPSC-CMs, APD_20_ and APD_50_ significantly shortened, but only when I_Kur_ was strongly increased (Figures 6A–D). APD_90_ was significantly reduced in ventricular-like, but not in atrial-like hiPSC-CMs (Figures 6E,F). APPlatA only showed a significant decrease in ventricular-like hiPSC-CMs (Figures 6G,H). Other AP parameters were unaffected upon increases in I_Kur_ (Figure 7).

## Discussion

Overall, the APs of our atrial-like hiPSC-CMs were substantially shorter and had a lower AP plateau than those of our ventricular-like hiPSC-CMs, in qualitative agreement with previous studies on atrial- and ventricular-like hiPSC-CMs (Marczenke et al., 2017; Verkerk et al., 2017; Argenziano et al., 2018; Cyganek et al., 2018; Lemme et al., 2018; Veerman et al., 2019). There are some quantitative differences in AP parameters with previous studies, but these are likely due to differences in cell lines, differences in differentiation protocols, absence or presence of I_K__1_ injection, and a different definition of AP plateau amplitude. The differences in AP parameters of our atrial- and ventricular-like hiPSC-CMs would have been even larger if we had supplied our atrial-like hiPSC-CMs with a more atrial-specific I_K__1_, as not only observed in human heart (Wang et al., 1998), but also in canine, murine and sheep heart (Dhamoon et al., 2004; Panama et al., 2007; Cordeiro et al., 2015). Of note, Fabbri et al. (2019) recently published a detailed *in silico* study of the effects of several I_K__1_ formulations on AP duration of hiPSC-CMs.

Maximum sustained native I_Kur_ density was larger in our atrial-like hiPSC-CMs than in our ventricular-like hiPSC-CMs. Yet, with a value of 1.88 ± 0.49 pA/pF at +50 mV, the I_Kur_ density of our atrial-like hiPSC-CMs was small in comparison with that of freshly isolated human atrial myocytes, for which Amos et al. (1996) observed a density of 5.1 ± 0.3 pA/pF for peak I_Kur_ and 4.7 ± 0.2 pA/pF for late I_Kur_ during a 300-ms voltage clamp step to +40 mV at 22°C (114 cells, 32 hearts). Therefore, we decided to block native I_Kur_ and use dynamic clamp to study the effects of I_Kur_, using a virtual I_Kur_ with characteristics, including its density, based on observations made in freshly isolated human atrial myocytes.

Due to the relatively low native I_Kur_ density of our atrial- and ventricular-like hiPSC-CMs, it was not surprising that blockade of I_Kur_ by 4-AP had only minor effects on AP parameters. When native I_Kur_ was replaced with virtual I_Kur_ with 100% density, similar to I_Kur_ density in freshly isolated human atrial myocytes, more pronounced effects on AP parameters were observed. In atrial-like hiPSC-CMs, APD_20_ shortened substantially, whereas APD_50_ shortened only moderately and APD_90_ even less so. In ventricular-like hiPSC-CMs, on the other hand, not only APD_20_, but also APD_50_ and APD_90_ shortened substantially upon injection of virtual I_Kur_. The more pronounced effect on APD in ventricular-like hiPSC-CMs is likely related to the longer and more positive AP plateau potentials leading to more functional consequences of I_Kur_. The observed decrease in APD_20_ was accompanied by a lowering of the AP plateau in both atrial- and ventricular-like hiPSC-CMs.

We only performed experiments at a pacing rate of 1 Hz and not at higher pacing rates. Therefore, we were unable to confirm that the relative contribution of I_Kur_ to AP repolarization increases with increasing pacing rate (Ford et al., 2016; Aguilar et al., 2017). Aguilar et al. (2017) carried out comprehensive computer simulations with the Courtemanche et al. (1998) human atrial myocyte model, in which the I_Kur_ formulation was updated in accordance with the experimental observations on I_Kur_ inactivation by Feng et al. (1998). They found that I_Kur_ did not inactivate significantly at high pacing rates and, consequently, the contribution of I_Kur_ to repolarization was mainly determined by its (fast) activation kinetics. Accordingly, rate-dependent changes in I_Kur_ were largely determined by changes in action potential morphology. In computer simulations with the Maleckar et al. (2009) model, on which we based our I_Kur_ formulation, we made similar observations (data not shown). We aim to test the rate dependence of the effects of I_Kur_ on AP repolarization in future experiments on hiPSC-CMs.

In both atrial- and ventricular-like hiPSC-CMs, simulation of loss-of-function mutations through lowering of the virtual I_Kur_ density from 100% to 12.5–75% of its control value, resulted in prolongation of the AP and raise of its plateau, in line with the differences in AP parameters that were observed between 0 and 100% I_Kur_. Marczenke et al. (2017) found that knock-out of *KCNA5* in hiPSC-CMs, representing a complete loss-of-function, may result in the development of EADs, which, however, were not observed in the present study, likely due to our higher pacing frequency. At I_Kur_ densities > 100%, simulating gain-of-function mutations, effects on AP parameters were somewhat less pronounced. In both atrial- and ventricular-like hiPSC-CMs, APD_20_ was only significantly decreased upon an increase in I_Kur_ density to 200%. A significant lowering of the AP plateau, together with AP shortening, was only observed in ventricular-like hiPSC-CMs.

Although the sustained native I_Kur_ density at +50 mV was small in our atrial-like hiPSC-CMs (1.88 ± 0.49 pA/pF, *n* = 17), it was still significantly larger than in our ventricular-like hiPSC-CMs (0.26 ± 0.26 pA/pF, *n* = 17). Within our atrial-like hiPSC-CM population we noted cells lacking I_Kur_ (Figure 4B), although atrial-like hiPSC-CM generation using retinoic acid has been shown to generate 90–95% atrial-like hiPSC-CMs (Cyganek et al., 2018), with the rest being sinus- or ventricular-like hiPSC-CMs. Since hiPSC-CMs display an immature phenotype, it is possible that not all atrial-like hiPSC-CMs have developed I_Kur_ densities large enough to be detected as 4-AP sensitive current in a voltage clamp setting. Our recorded I_Kur_ densities are lower than those of the only other known quantification of I_Kur_ density in atrial- and ventricular-like hiPSC-CMs (Kaplan et al., 2016). In the study by Kaplan et al. (2016), which has only been published in abstract form, the sustained I_Kur_ density at +50 mV amounted to 3.71 ± 0.55 pA/pF (*n* = 5) in atrial-like hiPSC-CMs, which was significantly larger than that of ventricular-like hiPSC-CMs (1.00 ± 0.10 pA/pF, *n* = 16). To distinguish between the two types of hiPSC-CMs based on I_Kur_ densities would require further investigation, although the available data suggest a trend of a significantly larger I_Kur_ density in atrial-like hiPSC-CMs.

Of note, all AP parameters of our atrial- and ventricular-like hiPSC-CMs except dV/dt_max_ and APA were not only different under control conditions, but also upon blockade of I_Kur_ by 4-AP, indicating that the two types of hiPSC-CMs are not only different in their level of K_v_1.5 expression, as determined by I_Kur_ density, and suggesting that differences in membrane currents other than I_Kur_ also contribute to the observed differences in AP parameters. This result is in line with previous findings by both Marczenke et al. (2017) and Lemme et al. (2018), who found that knock-out of *KCNA5* or I_Kur_ blockade by 4-AP in atrial-like hiPSC-CMs did not result in completely ventricular-like APs. These findings are, however, to some extent at odds with those by Kaplan et al. (2016), who noticed that the APs of their atrial-like hiPSC-CMs took on a ventricular-like shape when treated with 4-AP, which strongly suggested that I_Kur_ is the major determinant of atrial action potential morphology. Conversely, they observed that injection of a virtual I_Kur_ in ventricular-like hiPSC-CMs, employing the dynamic clamp technique using oocytes expressing a cloned K_v_1.5 current, resulted in APs similar to those of atrial-like hiPSC-CMs. Apart from the studies by Kaplan et al. (2016), Marczenke et al. (2017), and Lemme et al. (2018), data on I_Kur_ in hiPSC-CMs are limited and the electrophysiology of I_Kur_ in atrial- and ventricular-like hiPSC-CMs remains largely unknown.

Apart from demonstrating a link between altered I_Kur_ density and changes in AP parameters, I_Kur_ has now been quantified in both atrial- and ventricular-like hiPSC-CMs. Thus, the present study provides additional data toward a complete characterization of individual membrane currents in hiPSC-CMs. Moreover, our study illustrates the potentials of dynamic clamp experiments on hiPSC-CMs, allowing manipulation of characteristics of the injected current in real time, thus facilitating direct, systematic, and efficient testing of changes in those characteristics. In the context of studying drug effects, including effects of anti-AF drugs, dynamic clamp may prove useful in the identification of potential drug targets and in testing model-based hypotheses (Ortega et al., 2018). For instance, dynamic clamp experiments on atrial-like hiPSC-CMs with I_Kur_ based on specific loss- or gain-of-function mutations in *KCNA5* can be utilized to assess the cellular effects of these mutations as well as effects of dedicated pharmacological treatment through modulation of I_Kur_. Ultimately, this may lead to mutation-specific treatment of AF.

## Data Availability Statement

The raw data supporting the conclusions of this article will be made available by the authors, without undue reservation.

## Author Contributions

SH designed and performed the experiments, analyzed the data, and drafted the manuscript. HD cultured the hiPSC line and developed the procedures to generate atrial-like and ventricular-like hiPSC-CMs. LB prepared the hiPSC-CMs used for electrophysiology in the present study. AV and RW designed the study, interpreted the data, and drafted, edited, and approved the manuscript.

## Conflict of Interest

The authors declare that the research was conducted in the absence of any commercial or financial relationships that could be construed as a potential conflict of interest.
